# V-synthes2 - the Next Generation Tool for Structure-based Virtual Screening of Giga-scale Chemical Spaces

**DOI:** 10.21203/rs.3.rs-7782723/v1

**Published:** 2025-10-13

**Authors:** Antonina L. Nazarova, Anastasiia V. Sadybekov, Arman A. Sadybekov, Mykola Protopopov, Dmytro S. Radchenko, Yurii S. Moroz, Olga O. Tarkhanova, Vsevolod Katritch

**Affiliations:** University of Southern California; University of Southern California; University of Southern California; Chemspace LLC; Enamine Ltd; Enamine Ltd; Enamine Ltd; University of Southern California

**Keywords:** molecular docking, CADD, structure-based drug discovery, giga-scale chemical space, virtual ligand screening, open-source protocol

## Abstract

The recent advent of synthesizable on-demand chemical spaces of drug-like compounds opened new horizons in the discovery of ligands and drug candidates for clinically relevant targets, but exposed the scalability of computational screening as a key bottleneck. The modular V-SYNTHES approach has shown highly efficient > 1000-fold accelerated virtual screening, but its initial implementation was not fully automated, limited to the initial version of Enamine REAL space (11 billion), and its validation was limited to only two targets.

Here we present an upgraded V-SYNTHES2 workflow with improved automation features and scalability, expanded REAL Space of 36 billion readily available compounds, and assessing its performance on new, more challenging targets. As the original method, V-SYNTHES2 employs initial docking of the Minimal Enumeration Library (MEL) of fragments that represent all scaffolds and synthons of the REAL space. The best fragments are iteratively enumerated with corresponding synthons, and the intermediates redocked, until the fully enumerated molecules are docked and selected for synthesis. V-SYNTHES2 introduces a new geometry-based CapSelect method, allowing us to fully automate MEL fragment selection based on docking score and optimal binding pose. The method shows excellent enrichment and binding pose reproducibility in computational benchmarks, including challenging targets with shallow pockets, RNA-binding sites, G-protein-coupled receptors (GPCRs), and phospholipid-binding enzymes. Experimental testing shows the utility of this workflow in prospective screening campaigns for two new targets. The fully automated V-SYNTHES2 workflow (https://github.com/KatritchLab/V-SYNTHES2_pipeline/) can be deployed on computing clusters or clouds, offering a powerful tool for effective screening of giga-scale chemical spaces.

## INTRODUCTION

In recent years, computer-driven drug discovery has seen unprecedented growth, becoming mainstream in the drug discovery pipeline both in biopharmaceutical and academic settings^1,2^. This paradigm shift has been enabled by the availability of 3D structural information for clinically relevant targets by experimental techniques such as X-ray crystallography^3^, Cryo-EM^4^, as well as by AI-enabled modeling^5,6^. This shift is also supported by the growing computational resources and the rapidly expanding virtual chemical libraries and spaces^2^. Previously, the standard high-throughput (HTS) or Virtual Ligand Screenings (VLS) were largely limited to 1–10 million compounds available in diversity screening libraries or in stock from vendors^7,8^. The ultra-large on-demand virtual libraries of compounds like REadily AccessibLe (“REAL”) datasets of Enamine have dramatically increased the accessible drug-like space for screening^9^, showing utility in the discovery of initial hits and leads for major classes of targets, including enzymes^10, 11,12, 13^ and G-protein coupled receptors (GPCRs)^14,15,16,17^. Currently, Enamine REAL Space contains tens of billions of synthetically feasible compounds^18^, and is rapidly growing^19^. Compounds from both the fully enumerated and the non-enumerated REAL Space are synthesizable with an 80% success rate in a 4 weeks’ time frame, making them readily accessible for validation of virtual screening predictions and subsequent optimization of hits and leads.

With the rapid growth of the accessible drug-like space, the computational cost of virtual screening of tens of billions of drug-like compounds emerged as the key bottleneck in initial hit discovery. Several strategies to reduce this cost have been proposed, including fast GPU docking like AutoDock-GPU^20^, pipelines that funnel from fast and crude to more elaborate and refined docking^11,21^, or integration of physics-based methods like molecular docking with deep learning iterations^22, 23, 24, 25, 26, 27, 28^. Although capable of screening libraries of one or a few billion compounds on GPU clusters, these methods still require full library enumeration and pre-computing of ligand conformers, and thus face potential scalability issues with the continuing expansion of the accessible chemical space.

The general concept of Virtual SYNThon Hierarchical Enumeration Screening (V-SYNTHES^29^) approach, involves four iterative steps. (1) Generate a minimal enumeration library (MEL) of fragments that represent all possible synthon-scaffold combinations in the REAL Space, (2) Dock the MEL fragments in the target binding pocket and select a subset of the top highest-scoring and potentially productive MEL fragments, (3) enumerate synthons at the second attachment point of the subset fragments, and (4) re-dock this enumerated subset into the receptor. For those REAL Space molecules that contain 3 synthons, steps (3)-(4) are repeated until molecule completion. Fully enumerated molecules can be re-docked with more elaborate docking settings and further post-processed for the final candidate set selection^30^. The original V-SYNTHES 1.0 version, however, had several limitations, restricting its growth and performance. In V-SYNTHES 1.0, scaffold definitions were restricted to an early version of the REAL Space, encompassing 11 billion compounds. Additionally, the evaluation of MEL binding pose ‘productivity’ required users to define criteria for ‘productive’ poses for each target, precluding full automation.

**Figure 1.**
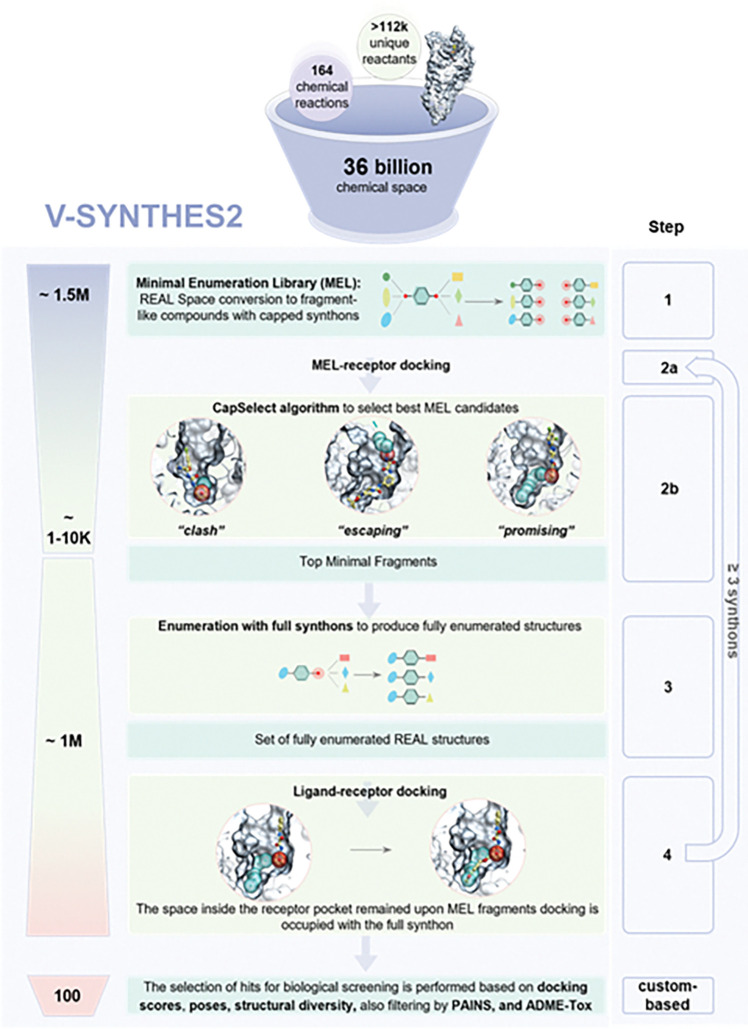
The V-SYNTHES2 workflow for screening Enamine’s REAL space: (1) Generation of minimally enumerated libraries (MEL) of fragments for 2-component, and 3-component reaction subsets of the REAL space; (2a) Docking of MEL fragments to the receptor pocket; (2b) Using the CapSelect algorithm to select MEL fragments that adopt productive poses within the receptor binding site; (3) Enumeration of the selected MEL fragments into fully or partially enumerated compounds; (4) Final docking of the fully or partially enumerated subset to the receptor. The last, postprocessing step involves the selection of potential hits from the top-ranked ~10,000 candidate set for their predicted physicochemical, ADME, and PK properties, novelty, and diversity.

In this study, we introduce V-SYNTHES2 (Fig. 1), an enhanced and fully automated platform expanding the efficient screening to Enamine REAL Space of 36 billion compounds. The newly developed approach to pose selection, CapSelect, automates the evaluation of ligand binding pose productivity by analyzing the geometry of docked MEL fragments within the target pocket, thereby streamlining one of the most complex steps in the screening workflow. Due to its iterative modular algorithm that does not require full enumeration of the whole space, V-SYNTHES2 computational demands scale linearly with the number of synthons, i.e. much slower than the total number of compounds in this space.

The initial computational benchmark shows that the pre-selection of MEL fragments with V-SYNTHES2 allows dramatic enrichment of high-scoring hits as compared to the standard VLS of random REAL Space subsets. Another key assessment introduced here shows high reproducibility of the docking poses between the initially docked MEL fragments and the matching moieties of the fully enumerated docked molecules, more than 90% for the 100K top-scoring molecules. This performance was achieved in a variety of target classes with challenging binding pockets characterized by shallow, small, and closed geometries. V-SYNTHES2 has been also applied in the prospective screening campaigns for two new challenging targets, phospholipase-class cPLA2 enzyme and angiotensin type 2 (AT2) receptor, validated experimentally in the accompanying studies^31,32^.

## Methods

### REAL Space and corresponding MEL fragments

The REAL Space of make-on-demand compounds, developed by Enamine, comprises billions of molecules synthesizable via optimized parallel synthesis protocols with a high success rate (> 80%) in 4 weeks^33^. The version of the REAL Space deployed here contains about 36 billion synthesizable molecules, involving 164 unique chemical reactions and 112,514 reactants. Enamine REAL Space consists of two types of compounds derived from (a) 2-component reactions, and (b) 3-component reactions. The reaction-related scaffolds are encoded in the Markush group format, featuring two, three, or four attachment points and corresponding R-group moieties denoting reaction synthons^18^. Each compound type is processed separately, resulting in two minimal enumeration libraries (MELs) for two-component or three-component reactions. These MEL compounds comprise a reaction scaffold with one attachment point fully enumerated with selected synthons from the REAL Space, while the other attachment point(s) are capped with minimal R-groups such as methyl or phenyl, depending on whether the reaction chemistry at those positions requires aliphatic or aromatic groups. For convenience, these capped R-groups are marked as radioisotopes.

**Figure 2.**
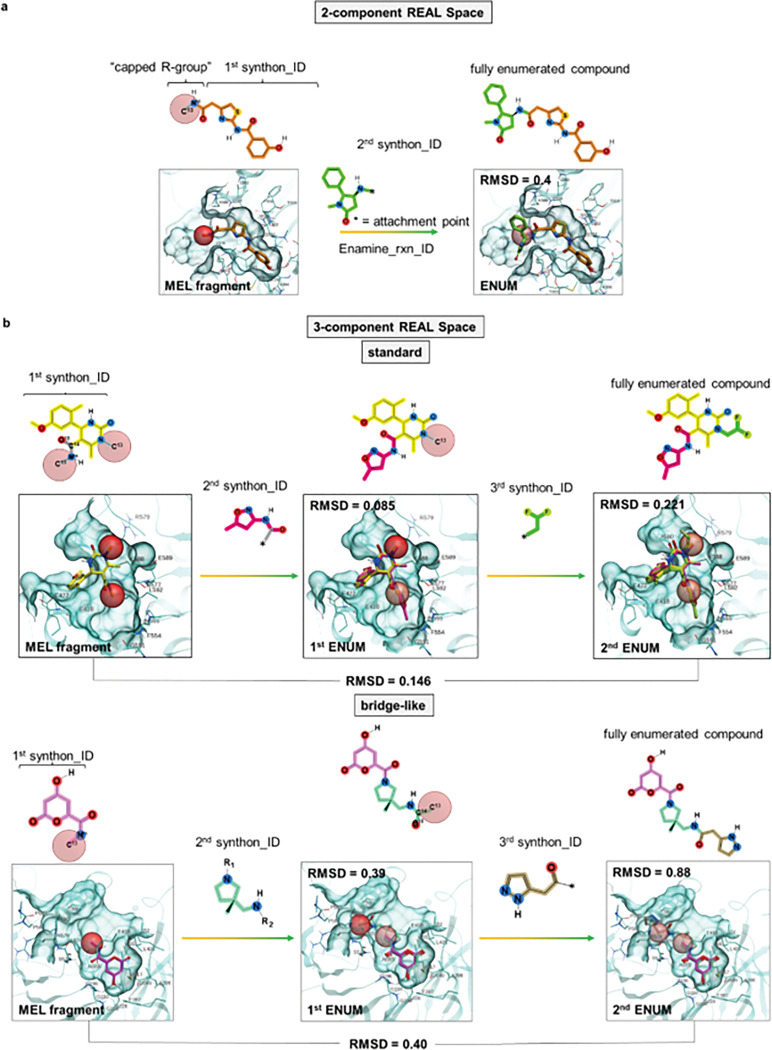
Enamine REAL Space of 36 billion compounds screening with V-SYNTHES2. (a) 2-component, and (b) 3-component reaction REAL Space subsets. The figure illustrates the binding poses of MEL and enumerated (for 3-component only) fragments, including synthons retained during enumeration, and minimal capped R-groups, which are converted to real synthons during the enumeration step. This example illustrates screening against the active site of the cPLA2 enzyme (PDB ID: 1CJY), with the ligand binding site shown in blue surface. Docked MEL fragments are shown as sticks, with synthon carbons color-coded to match the structural representation. Capped R-group carbons are shown as dark red spheres for the next enumeration stage and light red spheres for previous enumerations. Fragments are linked to specific IDs, which correspond to Enamine reaction IDs. Salt bridge interactions are depicted as black dashed lines, with labeled sticks representing specific residues within the binding pocket that interact with the binders. For this illustration, we presented cases with RMSD values matching the median RMSD on the set. For the 2-component reaction REAL Space, RMSD values are provided between MEL fragment and the first enumerated synthon (1st ENUM). For 3-component reactions, RMSD values are also given between MEL fragment and 1st ENUM, as well as between 1st ENUM and the fully enumerated molecules (2nd ENUM).

During the iterative enumeration process, the capped R-groups of the top fragments selected by docking MEL are replaced by the corresponding synthons from the library. For 2-component reactions, this enumeration is completed in one step. For 3-component reactions, two iterations are required to replace each of two capped R-groups with synthons from the REAL Space. Bridge-like 3-component reactions with consecutive connection of synthons requires special treatment during the enumeration step, as they are formed by a central bridge synthon connecting two terminal synthons. Bridge synthons by themselves usually lack specific pharmacophore groups and do not allow reliable docking, so they were not included in MEL library. The initial MEL fragments, therefore, contain only the terminal synthons capped by methyl R-group. The bridge synthon is added to the MEL fragment during the first iteration of enumeration, with the second side of the bridge synthon being capped by the methyl R-group. The second terminal synthon is enumerated in the second iteration, resulting in the fully enumerated molecule. For all types of reactions, this process ensures that the MEL fragment grows iteratively into fully enumerated molecules within the constraints of the REAL Space, while at each step maintaining chemical similarity to the final molecules and ensuring that top-scoring fragments are selected for enumeration (Fig. 2).

In the version of Enamine REAL Space described, the set of MEL fragments includes 1.7 million compounds for 2-component reactions, and 91.5K for 3-component reactions, which represents the full chemical diversity of the REAL Space. Note that because of different combinatorics in these two sets, the number of molecules in the fully enumerated REAL Space has a different trend: it has ~ 31.3 billion 3-component reaction compounds, and ~ 2.3 billion two-component reaction compounds. As the REAL Space continues to grow and expand with new reaction components, V-SYNTHES2 can be further modified to process the updated libraries, including compound with four or more components.

### V-SYNTHES2 Pipeline

The upgraded V-SYNTHES2 workflow involves the following steps shown in Fig. 1:

(1) Generate a minimal enumeration library (MEL) of fragments that represent all possible synthon-scaffold combinations in the REAL Space. In V-SYNTHES2, this step features a streamlined and automated MEL library preparation, specifically revamped to efficiently process the 36 billion compound REAL Space and its future expansions.
(2a) Dock the MEL fragments in the target binding pocket and (2b) select a subset of the top highest-scoring and potentially productive MEL fragments. In V-SYNTHES2, the CapSelect algorithm enhances MEL fragment selection by considering both docking scores and the predicted “productivity” of docking poses automatically using hybrid physics-geometry scoring-based algorithm, an improvement over the user-dependent definition of “productivity” in the first version. The CapSelect algorithm processes 30,000 MEL fragments in 10–20 minutes (depending on pocket geometry and the specific reaction-component subset of the REAL Space) using parallel computing across multiple CPU cores. It is effective for diverse binding pocket types—open, shallow, or closed—as validated in subsequent benchmarks. CapSelect operates with hyperparameters pre-optimized in initial studies and are embedded within the code, eliminating the need for target-specific adjustments. It selects productive fragments based on available sphere-based space and supports 4D docking models involving multiple receptor objects.
(3) Enumerate synthons at the second attachment point of the subset fragments. In V-SYNTHES2, for 3-component REAL Space, steps 2–4 must be repeated twice to fully enumerate compounds, while for the 2-component REAL Space, this process is done once.
(4) Dock the enumerated subset into the target binding pocket. The top-scoring subset of fully enumerated compounds (usually 10,000) is post-processed using additional scoring functions, filters for PAINS, physicochemical properties, drug-likeness, novelty, and clustering for chemical diversity (Fig. 1), resulting in 100–400 compounds selected for synthesis and testing.

V-SYNTHES2 operates on a distributed Linux computing environment, managed by shell-based job schedulers on computing clusters or cloud platforms. A typical screen can be completed in approximately 48 hours using 320 CPU cores per one reaction-based REAL subset. The final docking subset for all multicomponent libraries includes ~ one million compounds from productive MELs identified in CapSelect. Covering 2-component and 3-component reactions, V-SYNTHES2 requires docking of about two million fully enumerated compounds plus 1.8 million of MEL fragments, representing more than 10,000-fold reduction in computational resources over docking the full 36 billion REAL Space.

### CapSelect: MEL fragments with “productive” docking poses

In the initial applications of V-SYNTHES, it was observed that a capped MEL fragment’s docking pose, in addition to its docking score, can affect its “productivity,” referring to its potential to spawn high-scoring enumerated molecules. Fragments with sufficient pocket space at the capped attachment point allow for the expansion of capped R-groups during enumeration, while fragments with the capped R-groups confined to pocket “dead ends” are limited in their growth. In V-SYNTHES 1.0, the selection of productive fragments relied on user-specified unwanted contact residues, necessitating expert knowledge of the target pocket. V-SYNTHES2 addresses this with the CapSelect algorithm, an automated physics-based approach that evaluates the growth potential for each capped R-group in the docked MEL fragment.

**Figure 3.**
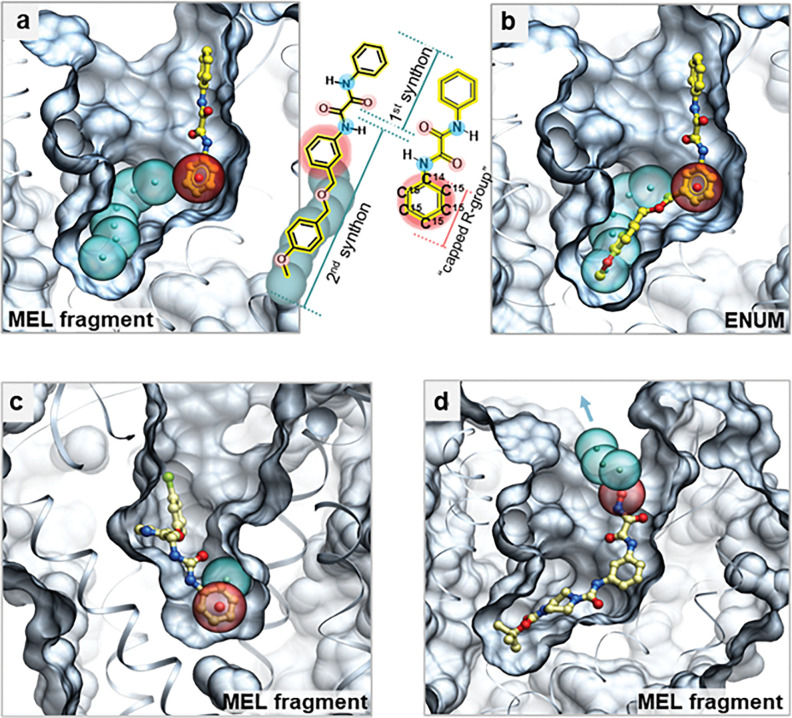
CapSelect’s Automated Selection of MEL Fragments in V-SYNTHES2 pipeline. Selection of optimal scaffold-MEL fragment combinations for extended molecule growth for 2-component reactions REAl Space subset. (a) the binding pose of a fully enumerated compound from the REAL space, produced in step (3) of the V-SYNTHES2 iteration. The calculated spheres predict the available space for accommodating a second synthon in the fully enumerated structure. Example of a deep pocket and open pocket (angiotensin receptor type 2 (AT2) (PDB ID: 6JOD); (a) CapSelect predicts the potential space within the binding site available after docking the primary 1^st^ synthon to accommodate a secondary 2^nd^ synthon (replacing the capped R-group, shown in red). This is depicted by a series of equidistant spheres (blue), illustrating a ‘productive’ outcome where the capped R-group is positioned to allow multiple sphere growth, indicating sufficient space for protein-ligand interactions upon synthon addition; (c) ‘non-productive’ outcome where the capped R-group (red, transparent) is near a pocket’s dead-end, permitting the growth of only one sphere (potentially accomodating single methyl group) (blue, transparent); (d) ‘non-productive’ outcome with the capped R-group positioned close to the pocket opening, leading to a suboptimal interaction between the receptor and the fully enumerated synthon compound.

CapSelect, illustrated in Fig. 3, predicts the growth capacity of docked MEL fragments at attachment points during synthon enumeration. The process begins with a sphere placed at the labeled (“radioactive”) carbon of the capped R-group (Fig. 3a, b). Subsequent non-overlapping spheres of a fixed diameter are generated iteratively to avoid intersecting the pocket or ligand. Each next sphere centroid is selected in the 120-degree cone defined by the vector connecting the previous centroids, so that the centroid distance to the pocket atoms is maximized. This algorithm uses spherical meshes, constructed with 72 azimuthal and 36 longitudinal angles in 5° increments for optimal balance of geometric detail and computational efficiency.

Sphere radii were set at 2 Å, accounting for the typical C(sp3)-C(sp3) bond length (~ 1.54 Å) and the surrounding atomic environment and functional groups. The initial capped R-group sphere radius is 3.5 Å for phenyl-based capped R-group and 3 Å for methyl-based capped R-groups, values empirically optimized for a variety of targets. The iterative placement of spheres ensures 2 Å spacing from pocket-lining atoms. Although mesh resolution can be adjusted in the source code, modifications are not recommended, as optimal hyperparameters have already been established (https://github.com/KatritchLab/V-SYNTHES2_pipeline/).

The iterative generation of spheres in CapSelect stops when either the next attempted sphere clashes (i.e. cannot be placed at > 2Å distance from atoms of the pocket or ligand itself) or the sphere chain extends too far beyond the pocket (> 10Å).

The CapScore is then calculated to evaluate the MEL fragment productivity, taking into account spheres fitted within the pocket before steric crowding or before exiting the pocket. For standard 3-component reactions, CapSelect incorporates additional criteria for MELs with two capped R-groups. When dual growth pathways are available, the more favorable pathway is selected. Fragments are classified as unproductive if both R-groups encounter steric blocks or dead ends (Supporting Information, Figures SI 1a, 1b). Conversely, fragments remain productive if at least one R-group supports continued growth while the other does not (Figures SI 1c, 1d).

The CapScore and Docking Score are integrated via the MergedScore function, a weighted logarithmic sum of these scores detailed in Supporting Information, Table 1. The Docking Score, representing the strength of ligand binding, is provided by the ICM docking procedure. The CapSelect algorithm ranks MEL components by descending MergedScore, sequentially enumerating MELs to generate one million compounds from REAL Space. This typically requires fewer than 1,000 fragments for two-component reactions, and up to 45,000 for 3-component subsets, depending on receptor geometry and the REAL Space version. Supporting Information (Table SI2) provides MEL fragment counts needed for one million partially or fully enumerated compounds.

Supporting Information, Figure SI2 shows Docking Score vs. CapScore parity plots for cPLA2 (shallow pocket) and RhoR (closed pocket), highlighting top MELs selected by CapSelect compared to those chosen solely by docking scores. Overlap between the 2-component MEL candidates selected for enumeration by Docking Score and CapScore was 83% for cPLA2 and 48% for Rho. For other binding pocket geometries, such as deep and narrow pockets or shallow pockets at monomer interfaces, the overlap between MEL fragments selected by CapSelect and those with top docking scores varies. It reaches nearly 70% for the angiotensin receptor type 2 (AT2R) and up to 93% for the transient receptor potential cation channel subfamily V member 2 receptor (TRPV2R) target (Supporting Information, Figure SI2 c and d).

### V-SYNTHES2 workflow

The workflow was optimized for automated virtual screening on multi-CPU HPC systems and deployed on a Slurm-managed Linux cluster. While all stages were automated for cluster execution (in the USC CARC environment, docking jobs were executed with the Slurm workload manager on the epyc-64 partition with AMD EPYC 7513 CPUs (2.60 GHz, 64 CPU cores per node, 256 GB memory per node, 78 nodes), which supported up to 1,200 concurrent CPU jobs, 5,000 queued jobs, and a maximum runtime of 48 hours), CapSelect (Stage 2b), enumeration (Stage 3), and postprocessing were executed locally on a high-performance workstation with fast storage and a multi-core CPU environment. Smaller sets were processed on the main partition for serial and moderately parallel jobs (on average, chunks of 50,000 fully enumerated molecules required 10–30 hours to be docked using 16 cores per job). In the current V-SYNTHES2 version, the screening of the dataset of 36 billion compounds requires about 38,400 CPU hours (or about $300 at $0.01/CPU hour on the cloud). The actual processing times can range from hours to days, depending on computational resources and the number of CPUs used. Typically, we employ ~ 400 CPUs per REAL subset project, completing it in ~ 48 hours. Recently, V-SYNTHES2 was tested using experimental xREAL Space with 173 billion compounds which required only a minor increase in CPU cost, keeping it affordable. However, further scaling beyond trillions of compounds may require more significant computing costs in order to maintain reliable retrieval of most high-quality hits. The future growth should be accommodated by the development of additional accelerators, e.g., based on Deep Learning.

Stage 1 of protein grid preparation and final selection of the top hits (typically 100–200 compounds) was performed using a graphical user interface (GUI) to visually inspect binding poses and confirm key interactions within the binding site. RMSD calculations between MEL fragments and fully enumerated molecules were run locally or in parallel on a multi-CPU workstation, and Python scripts supported RMSD analysis, including median, dispersion, and interquartile range, which were visualized via violin plots.

Molsoft ICM-Pro v. 3.9–2b was used as a platform, including imbedded scripting language, cheminformatics tools, and docking procedure. In addition, C + + environment was used (executables for Ubuntu 20.04 are provided); and a Python 3.0 + conda environment with RDKit, pandas, and numpy was used for RMSD assessment between MEL fragments and fully enumerated molecules after V-SYNTHES2.

### Receptor and ligand preparation

Receptor and ligand preparation, including docking grid map generation, protonation, charging of the MEL and synthon libraries, and 3D conformer generation for fully enumerated molecules—was performed in the ICM-Pro v3.9–2b molecular modeling suite from Molsoft LLC^34^. Docking models were based on receptor structures obtained from the PDB database^35^. The Rho receptor (opsin receptor family) was modeled based on its inactive-state bovine X-ray structure (PDB ID: 1U19, resolution 2.2 Å), co-crystallized with the small-molecule chromophore 11-cis-retinal and associated water molecules^36^. The AT2 receptor (angiotensin receptor family) was modeled using its active-state human X-ray structure complexed with the angiotensin II peptide (PDB ID: 6JOD, resolution 3.2 Å)^37^. The cPLA2 enzyme (phospholipase family) was modeled from its apo-form X-ray structure (PDB ID: 1CJY, resolution 2.5 Å)^38^. Non-essential components such as non-structural water molecules, protein-stabilizing single-chain antibodies, and other non-essential molecules were removed, leaving only the receptor protein subunit(s) (and in some cases important cofactors like ions and tightly bound water molecules defining the target binding pocket). Each model was then processed by adding and optimizing hydrogen atoms and refining side chain residues with the co-bound ligand, considering correct chiral definition and formal charge assignment. This process involved iterative rounds of minimization and Monte Carlo conformational sampling of the ligand and side chain residues within 5–8 Å of the binding site. For a selected all-atom model, the grid potentials were then calculated within a box specified by the user around a proposed ligand binding pocket, giving sufficient margins to accommodate all possible ligand poses and interactions within the box. Although docking into a box that covers the whole receptor was possible, selecting a smaller box speeded up and improved the convergence of sampling. Grid potentials were calculated for electrostatic, hydrophobic, hydrogen bonding, and van der Waals energy terms, taking into account some receptor flexibility through the use of “soft” van der Waals potentials. To accommodate more pronounced flexibility, grid potentials of multiple conformations of the binding pocket were merged into 4D docking models^39^, or explicitly flexible side chains of the pocket can be specified^40^.

V-SYNTHES2 workflow scripts (ICM scripts, C + + code, and bash scripts), as well as ICM, Python, and bash scripts for RMSD assessments between MEL fragments and enumerated molecules, were uploaded to https://github.com/KatritchLab/V-SYNTHES2_pipeline/; a text editor was used as needed. A single V-SYNTHES2 run encompassing the 2-component and 3-component REAL Spaces required 4–40 GB of storage, depending on the number of molecules saved after docking.

## RESULTS and DISCUSSION

### Reproducibility of MEL fragment binding poses in fully enumerated REAL compounds

**Figure 5:**
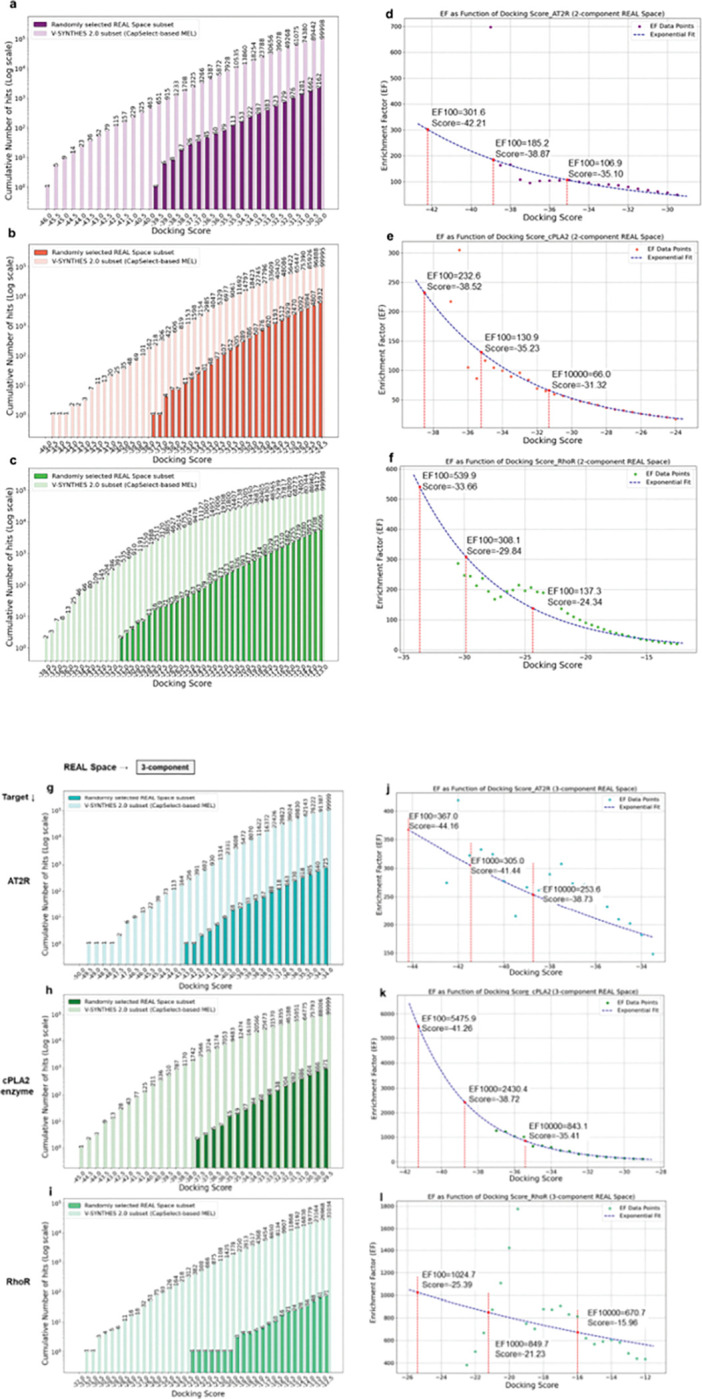
Figure 6. Enrichment of high-scoring compounds in V-SYNTHES2 fully enumerated pool compared to random property matched subset of REAL Space: The subset enumerated by V-SYNTHES2 was derived from MEL fragments selected via the CapSelect approach for (a,d,g,j) AT2 receptor; (b,e,h,k) cPLA2 enzyme; (c,f,I,l) Rho receptor targets. Cumulative histograms a,b,c,g,h,i) display the number of hits on a logarithmic scale against the docking score threshold for both the V-SYNTHES2 subset and the REAL random subset. Red vertical lines indicate 100, 1000, and 10,000 V-SYNTHES hits. Scatter plots (d,e,f,j,k,l) illustrate the enrichment in hit identification performance of V-SYNTHES2 compared to conventional VLS. Enrichment factors for 100, 1000, and 10,000 virtual hits (EF10,000) are highlighted with stars. Note: V-SYNTHES 1.0 evaluated only EF100.

One of the key internal controls for V-SYNTHES algorithm performance is that the binding poses of the top-ranking MEL fragments are effectively reproduced in the docking of corresponding top-ranking fully enumerated molecules. We performed RMSD analysis to compare the binding poses of MEL fragments and their corresponding moieties in fully enumerated molecules across the 2-component, and 3-component reaction subsets of REAL Space in V-SYNTHES2. Binding pose reproducibility was assessed for the orthosteric binding sites of three receptors: (a) the deep and narrow pocket of the AT2 receptor, (b) the shallow binding pocket of cPLA2, and (c) the small, enclosed pocket of the rhodopsin receptor. The fragment docking pose is expected to be reliably reproducible only for high-scoring candidate hit compounds, while reproducibility can fade for low-scoring compounds that are not predicted to be specific binders. Therefore, we assessed the top 1K, 10K and 100K highest scoring hits (Supporting Information, Table SI 3). For the 2-component subset, RMSD was measured between MEL fragments (excluding radioactive atoms in capped R-groups) and the corresponding moieties in the enumerated molecules (Supporting Information, Figures SI 3, SI 5, SI 7). For 3-component subset, RMSD was calculated in three ways: (1) between MEL fragments and the partially enumerated molecules (1st ENUM), (2) between the partially and fully enumerated (2nd ENUM) molecules, and (3) between MEL fragments and the final fully enumerated molecules (Supporting Information, Figures SI 4, SI 6, SI 8). Figure 5 presents the statistical RMSD analysis for the top 10K hits. For 2-component libraries, all three pockets exhibited excellent reproducibility of the initial docking poses, with median RMSD values below 0.5 Å across the top 10K ranked sets. In the 3-component subset, cPLA2 demonstrated the lowest median RMSD (0.17 Å), while the AT2 receptor displayed higher median RMSD (0.68 Å). This increase is primarily due to the presence of secondary peaks observed in many RMSD distributions, associated with compound flipping, wherein molecules adopt multiple binding orientations with comparable docking scores. Pose reproducibility can vary more substantially in three-component libraries due to the typically smaller size of the initially enumerated synthon, resulting in a less reliable anchoring fragment. Consequently, in the case of Rho, which possesses a very small and enclosed binding pocket, the median RMSD between the MEL-generated and fully enumerated ligand sets reached approximately 4.65 Å, reflecting the impact of the secondary distribution peak. To benchmark CapSelect impact on pose reproducibility, we compared CapSelect-based fully enumerated subsets, with a baseline strategy referred to here as ‘greedy’, which focuses on selecting MEL fragments solely on docking scores (Supporting Information, Table SI 3; Supporting Information, Figures SI 3–8). CapSelect significantly outperformed the greedy method in reducing RMSD across various subsets, proving that CapSelect successfully selects fragments with poses favorable for further growth of the molecule in the binding site. Essentially, because CapSelect predicts and removes MEL fragments with docking poses not compatible with enumeration, it reduces those cases when the scaffold is forced to flip due to steric clashes introduced by replacement of cap with bulkier REAL synthons. The observed differences in mean values between MEL and first enumeration, notable in the 3-component reaction RMSDs, arise because CapSelect was not applied prior to the initial enumeration step, leading to some scaffold molecules undergoing orientation changes (flipping). This underscores the strength and utility of CapSelect, highlighting its capability to maintain consistent scaffold orientation by preemptively selecting compatible fragments, thereby reducing unwanted conformational changes. For the AT2 receptor, CapSelect achieved a 1.5-fold reduction in both median and interquartile range of RMSD within the top 10K scored compounds in the 2-component subset. For the 3-component reaction subset, RMSD reductions were comparable for cPLA2 and Rho targets. However, for AT2R, the CapSelect-based subset exhibited an almost twofold decrease in interquartile range compared to the greedy-based subset, indicating a lower proportion of flipping, highlighting the superior precision of CapSelect. When evaluating the percentage of compounds with RMSD values below 3 Å relative to their corresponding MEL, the 2-component REAL space showed approximately 90% consistency across all three targets. In the case of 3-component compounds, the consistency was approximately 70% for AT2 and around 90% for cPLA2 at both stages of enumeration. However, for the Rho target, this consistency dropped to nearly 40% due to the limited size of its binding pocket, causing the larger 3-component compounds to adopt alternative orientations.All the screening statistics confirming the successful completion of all docking jobs executed during V-SYNTHES2 benchmarking, including 2-component, 3-component reaction sets screened against AT2, CB2, and cPLA2 targets is reported in Table SI 5.

### Performance of V-SYNTHES2 in iterative enrichment of high-scoring compounds

Another important internal assessment calculates the enrichment factor (EF), i.e. the ability of V-SYNTHES steps 1–3 to enrich the final enumerated set with high-scoring compounds as compared with a randomly chosen property-matched REAL Space subset of the same size. While in the original V-SYNTHES 1.0 study^29^ we calculated EF factors at the score threshold corresponding to the best 100 compounds (EF_100_), here we expanded the assessment to score threshold 10,000 (EF_10,000_) hits. The EF_10,000_ values, although generally lower than EF_100_ provide more statistically reliable estimates, especially as enrichments grow with the size of the REAL Space screened (Supporting Information, Table SI 4). The details of EF assessment are described in the Procedure Stage VIII: Random Sampling of the REAL Space for Statistical Analysis of Enrichment Values of Procedure I.

For the two-component subset, V-SYNTHES2 demonstrated EF_10,000_ values of 107, 71, and 137 for the AT2, cPLA2, and Rho receptors, respectively (Fig. 6 and Supporting Information, Figures SI 9, 11, and 13). In 3-component reactions, the enrichment factor substantially improved across all three targets, achieving EF_10,000_ values of 107 for AT2, 1270 for cPLA2, and 671 for Rho (Supporting Information, Figures SI 10, 12, and 14). Comparisons between CapSelect-based and greedy-based selections in the two-component subset indicated that CapSelect provided a 1.5-fold improvement in EF_10,000_ for Rho, and improvements of 1.2-fold and 1.4-fold for AT2 and cPLA2, respectively (Supporting Information, Table SI 4). For 3-component reactions, CapSelect yielded a 1.3-fold higher EF_10,000_ for the closed Rho binding pocket, yet no improvement for the open and shallow cPLA2 pocket, and structurally well-defined, recessed AT2 pocket.

Additionally, CapSelect was benchmarked against a manual selection process for the cPLA2 receptor, which followed V-SYNTHES 1.0’s guidelines^29^, based of distance constraints to avoid binding dead-ends. While the manual selection outperformed a random subset with an EF_10,000_ of 24 (Supporting Information, Figure SI 11(c)), the CapSelect approach yielded almost tripled the high-scoring hits, highlighting its efficiency in selecting productive MELs in terms of both time and results (Supporting Information, Table SI 4).

### Applications to specific protein targets

V-SYNTHES2 was applied to screen giga-scale REAL Space for various challenging targets, most recently to angiotensin type 2 (AT2) receptor^31^, and cytosolic phospholipase A2 (cPLA2) enzyme^41^. The distinct binding pocket geometries of these receptors made them especially suitable targets for validating the V-SYNTHES2 platform. Thus, cPLA2 has a shallow ligand-binding active pocket, the structure of which was determined experimentally only in the apo state^38^. Another target was angiotensin receptor AT2, a peptide-activated GPCR, for which the discovery of selective small-molecule agonists is considered a major challenge. Prospective screening of REAL for both models was performed using the methods and models of cPLA2 and AT2 described and computationally benchmarked in this study. The postprocessing results for 10,000 top-scoring compounds from V-SYNHTES2 screenings for AT2 and cPLA2 are given in Table SI 6).

As described in detail in ref^41^, the experimental validation of V-SYNTHES2 predictions for cPLA2 revealed that 19 out of 117 synthesized and tested ligands show significant (> 40%) inhibition at 10 μM (16% hit rate). SAR-by-catalog and another round of optimization of the two best hits resulted in potent compounds BRI-50460 and BRI-50469 with improved solubility, bioavailability, and brain permeability. Further in vitro testing of BRI-50460 showed its high potency at cPLA (0.88 nm) and selectivity against iPLA homolog. In vivo testing of BRI-50460 demonstrated favorable brain-to-plasma ratios and its ability to modulate neuroinflammatory pathways by restoring lipid homeostasis. In cultured astrocytes and neurons derived from human induced pluripotent stem cells, BRI-50460 mitigated the effects of amyloid beta 42 oligomers on cPLA2 activation, tau hyperphosphorylation, and synaptic and dendritic reduction. These results support BRI-50460 as a potential lead candidate for the treatment of neuroinflammation in Alzheimer’s disease and other neurodegenerative disorders^41^.

Another application targeted the identification of agonists for angiotensin type 2 (AT2) receptor in the assays measuring nitric oxide (NO) release levels in cell, identifying two new agonists out of 40 tested with significant activity at 1 μM concentration^31^. SAR-by-catalog and optimization of these hits is ongoing, with potential therapeutic applications for conditions such as hypertension and cardiovascular diseases^31^.

## Supplementary Material

Supplementary Files

This is a list of supplementary files associated with this preprint. Click to download.

• VSYNTHES2SISubmitted.docx

## Figures and Tables

**Figure 4:**
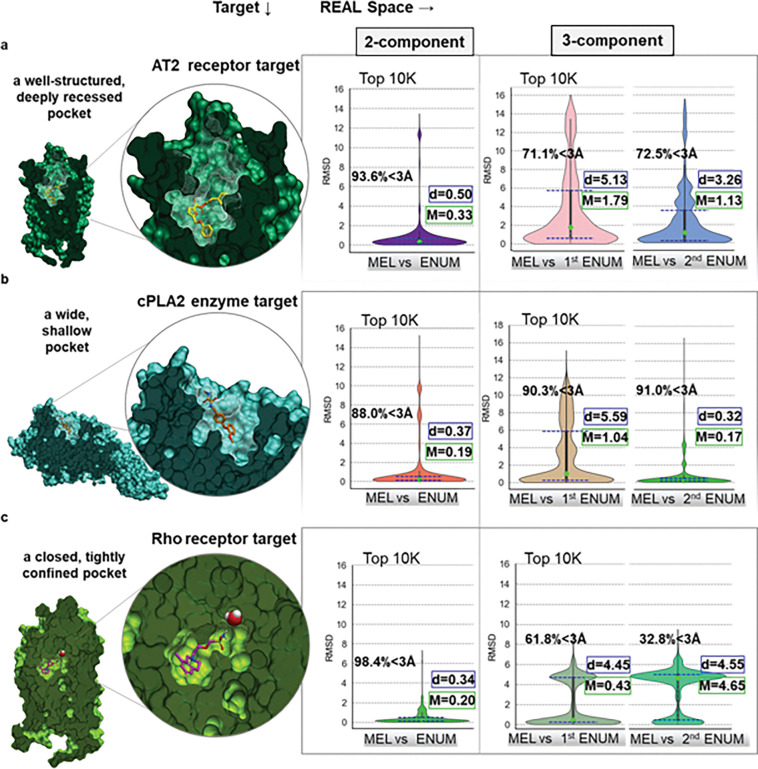
Figure 5. Assessment of reproducibility of MEL fragment binding poses with the corresponding enumerated molecules in the 2-component, and 3-component reaction subsets of V-SYNTHES2. Statistics are presented for the first 10K compounds with the highest docking scores across screening campaigns for (a) the AT2 receptor, (b) the cPLA2 enzyme, and (c) the Rho receptor. Promising MEL fragments were selected using a CapSelect-based strategy for the 2-component and subsequent stages of 3-component REAL subsets. For stage 1 screening in the 3-component REAL subset, productive MEL fragments were chosen based solely on their docking scores. The lime dot in each plot indicates the median (M), while the blue dashed lines represent the quartiles, with the interquartile distance denoted as d and the percentage of endpoints with RMSD<3Å.

## Data Availability

The prepared recent version of the REAL Space should be requested from Enamine at libraries@enamine.net

## Code availability

V-SYNTHES2 is developed using a combination of programming languages, including Python, C++, Molsoft ICM scripting, and bash scripting. ICM scripting is the language used in the ICM-Pro software environment. The software’s workflow is organized in the V-SYNTHES2 pipeline, where the execution sequence is defined as a series of shell commands. The repository for CapSelect, which includes both Python and C++ versions, along with a Shell-based multiprocessing module and two ICM scripts for input generation, is accessible at: https://github.com/KatritchLab/V-SYNTHES2_pipeline/CapSelect. The entire codebase for V-SYNTHES2 is freely available at https://github.com/KatritchLab/V-SYNTHES2_pipeline/ under the GNU GPL open-source license. This repository provides comprehensive instructions for running V-SYNTHES2, and practical examples.
